# Genotype Profile of Global EYS-Associated Inherited Retinal Dystrophy and Clinical Findings in a Large Chinese Cohort

**DOI:** 10.3389/fcell.2021.634220

**Published:** 2021-06-11

**Authors:** Ke Xu, De-Fu Chen, Haoyu Chang, Ren-Juan Shen, Hua Gao, Xiao-Fang Wang, Zhuo-Kun Feng, Xiaohui Zhang, Yue Xie, Yang Li, Zi-Bing Jin

**Affiliations:** ^1^Beijing Ophthalmology and Visual Science Key Laboratory, Beijing Tongren Eye Center, Beijing Tongren Hospital, Beijing Institute of Ophthalmology, Capital Medical University, Beijing, China; ^2^School of Ophthalmology and Optometry, The Eye Hospital, Wenzhou Medical University, Wenzhou, China

**Keywords:** inherited retinal dystrophy, copy number variations, mutation spectrum, hot spot mutation, genotype-phenotype trait, EYS mutation

## Abstract

**Purpose:**

The aim of this study was to probe the global profile of the EYS-associated genotype-phenotype trait in the worldwide reported IRD cases and to build a model for predicting disease progression as a reference for clinical consultation.

**Methods:**

This retrospective study of 420 well-documented IRD cases with mutations in the *EYS* gene included 39 patients from a genotype-phenotype study of inherited retinal dystrophy (IRD) conducted at the Beijing Institute of Ophthalmology and 381 cases retrieved from global reports. All patients underwent ophthalmic evaluation. Mutations were revealed using next-generation sequencing, followed by Sanger DNA sequencing and real-time quantitative PCR analysis. Multiple regression models and statistical analysis were used to assess the genotype and phenotype characteristics and traits in this large cohort.

**Results:**

A total of 420 well-defined patients with 841 identified mutations in the *EYS* gene were successfully obtained. The most common pathogenic variant was a frameshift c.4957dupA (p.S1653Kfs^∗^2) in exon 26, with an allele frequency of 12.7% (107/841), followed by c.8805C > A (p.Y2935X) in exon 43, with an allele frequency of 5.9% (50/841). Two new hot spots were identified in the Chinese cohort, c.1750G > T (p.E584X) and c.7492G > C (p.A2498P). Several EYS mutation types were identified, with CNV being relatively common. The mean age of onset was 20.54 ± 11.33 (4–46) years. Clinical examinations revealed a typical progression of RPE atrophy from the peripheral area to the macula.

**Conclusion:**

This large global cohort of 420 IRD cases, with 262 distinct variants, identified genotype-phenotype correlations and mutation spectra with hotspots in the *EYS* gene.

## Introduction

Inherited Retinal Dystrophy (IRD) includes a variety of genetic retinal disorders linked with disease-causing mutations. It is characterized by progressive retinal degeneration and photoreceptor loss (Bernardis et al., 2016; Broadgate et al., 2017), and has a global prevalence rate of 1 in 2000–3000 (Hartong et al., 2006). The primary form of progressive IRD is retinitis pigmentosa (RP) (OMIM #268000), with a global incidence of ∼1 in 4,000 births and 2.5 million affected patients (Hartong et al., 2006; Dias et al., 2018). RP is a highly heterogeneous genetic disorder in humans (Bandah-Rozenfeld et al., 2010), with more than 100 causative genes identified (a detailed list is available at the RetNet database^1^ and the RetinoGenetics database^2^) (Ran et al., 2014). RP shows a significant genetic heterogeneity, with multiple inherited patterns including autosomal recessive (AR), autosomal dominant (AD), and X-linked (XL) forms (Bunker et al., 1984; Grøndahl, 1987).

Mutations in the eyes shut homolog (*EYS*) gene, one of the most prevalent causes of RP, account for about 5–10% of ARRP (Audo et al., 2010; Barragán et al., 2010; Littink et al., 2010; Hosono et al., 2012). The *EYS* mutations were first reported as a genetic cause of ARRP in 2008 (Abd El-Aziz et al., 2008; Collin et al., 2008). The *EYS* gene has 43 exons and is located on chromosome 6q12. Spanning approximately 2 MB, this gene is one of the largest genes identified in the human genome (Abd El-Aziz et al., 2008; Barragán et al., 2010; Littink et al., 2010). The gene encodes a protein located in the outer segment of the photoreceptor cells (Abd El-Aziz et al., 2008) and is an ortholog of the Drosophila spacemaker (spam) protein. The protein is encoded by the longest isoform of the gene, containing 3165 amino acids, and is subdivided into 28 epidermal growth factor-like (EGF) domains and 5 Laminin A G-like domains at the C-terminus (Abd El-Aziz et al., 2008; Bandah-Rozenfeld et al., 2010; Barragán et al., 2010). Functionally, the EYS protein is indispensable for sustaining the morphological stability of the photoreceptor (Abd El-Aziz et al., 2008). However, the exact pathogenic role of the protein requires further elucidation.

The large number of EYS mutations reported to date in many independent studies prompted the present study aimed at assessing the effects of each mutation. The correlations between genotype and phenotype have not yet been well defined in patients with EYS mutations. We included 420 participants with *EYS* mutations in the present study to explore the possible correlations between genotypes and clinical phenotypes. Our findings provide a global profile of *EYS*-associated genotype-phenotype traits in patients with IRD and a model for predicting the progression of the disease as a reference for clinical consultation.

## Subjects and Methods

### Study Subjects

The study was conducted in compliance with the protocols of the Medical Ethics Committee of Beijing Tongren Hospital and with the tenets of the Declaration of Helsinki. We classified 39 unrelated patients with *EYS* gene variants who had a diagnosis of *EYS*-IRD as group one (G1). This group included nine probands with family histories and 30 sporadic cases, making it the largest cohort of *EYS* patients with genetic and clinical information in Asia to date. Every study participant provided informed consent for the clinical and genetic studies. All patients’ medical and family histories were taken carefully; these histories mainly covered the chief complaints of the initial ocular symptoms (such as low vision or night blindness), onset age of the disease, and clinical information about their available relatives. Ophthalmic examinations, including best-corrected visual acuity, slit-lamp biomicroscopy, subjective and objective optometry, and fundus photography, were performed for each participant. Some patients also underwent optical coherence tomography (OCT), full-field electroretinography (ERG), and autofluorescence imaging (AF).

We also retrospectively investigated 381 previously reported index probands from various populations who carried *EYS* mutations and included their available clinical information published until June 2020; these patients were classified as group two (G2). Detailed literature sources for the G2 group variants are provided in Supplementary Table 1. In brief, our study included 420 patients divided into two cohorts.

### Targeted Exome Sequencing and Bioinformatics Analysis

Peripheral venous blood samples of each participant and their available family members were collected. Genomic DNA was extracted from the peripheral leukocytes according to standard extraction protocols using the Whole Blood Genomic DNA Extraction Kit (Vigorous, Beijing, China). The DNA concentration was quantified with a NanoDrop 2000 device (Thermal Fisher Scientific, DE) (Chen et al., 2020).

The participants’ genetic information was analyzed with subsequent targeted exon sequencing (TES) to develop a sequencing panel including 188 known inherited retinal degeneration (IRD) genes (Sun et al., 2018). The Illumina library preparation, capture experiments, sequencing raw data analysis, and subsequent bioinformatics evaluation have been described previously (Sun et al., 2018). Multiple databases were used for annotation and evaluation of the variants, and the pathogenicity of missense variants was predicted *in silico* by Mutation Taster, PolyPhen-2, and SIFT. Variants with a potential splicing effect were predicted based on their ada-boost and random forest scores. We appraised the sequence variant classifications based on the American College of Medical Genetics and Genomics (ACMG) policies and guidelines (Richards et al., 2015). The HGMD database was used to query reported pathogenic mutations. Real-time quantitative PCR was performed for three probands to confirm the presumed copy number variations (CNV) of *EYS*. All the putative pathogenic EYS variants (NM_001142800) were validated using Sanger sequencing. The available family members were evaluated for co-segregation.

### Statistical Analysis

Statistical analyses were performed using SPSS version 22.0 software (IBM, Corp., Armonk, NY, United States). The best corrected visual acuity (BCVA) was converted to the logarithm of minimum angle of resolution (logMAR) unit for statistical analysis. Logistic regression analysis was performed to evaluate the visual progression by calculating the probability of low vision or blindness observed at different ages. The WHO criteria were used to set low vision and legal blindness as 0.05 ≤ BCVA < 0.3 and BCVA < 0.05, respectively. A *p*-value < 0.05 was considered statistically significant. The Kolmogorov-Smirnov test was performed to assess the normal distribution of visual acuity. The Wilcoxon Rank Sum Test was performed to evaluate the difference between genotypes.

## Results

### Molecular Diagnosis in EYS Gene

**FIGURE 1:**
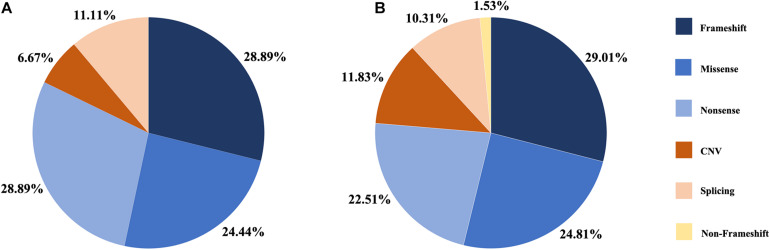
Distribution of the variant types in this study. **(A)** Proportion of variant types in G1 cohort. **(B)** Proportion of variant types in two cohort, G1 and G2.

In the G1 cohort, we identified 45 distinct causal or likely causal variants, including 11 missense, 13 nonsense, 13 frameshift, 5 splice-site, and 3 CNV mutations (Figure 1A). Of these 45 mutations, the most prevalent was c.6557G > A (p.G2186E), identified 7 times with an allele frequency 9% (7/78), followed by c.1750G > T (p.E584X) and c.7492G > C (p.A2498P) with allele frequencies of 6.4% (5/78), and variants c.7228 + 1G > A and c.8805C > A (p.Y2935X) with allele frequencies of 5.1% (4/78). This was the first report of 19 of these 45 variants and included 4 new missense, 7 new nonsense, 6 new frameshift, and 3 new CNV mutations. Two novel missense variants, c.359C > G (p.T120R) and c.9002C > T (p.A3001V), were identified in two unrelated probands. The *in silico* analysis programs of SIFT showed that these mutations could be damaging, while the Mutation Taster and Polyphen2 programs indicated they were likely benign. The frequency of variant p.T120R was less than 0.005 in database ExAC and gnomAD, and the frequency of variant p.A3001V was not recorded in any public databases; therefore, according to the ACMG guidelines, the classification of these two mutations is uncertain. Detailed results of the frequency and pathogenicity analyses of G1 group variants are presented in Supplementary Table 2. Cosegregation analysis was performed in 39 families.

**FIGURE 2:**
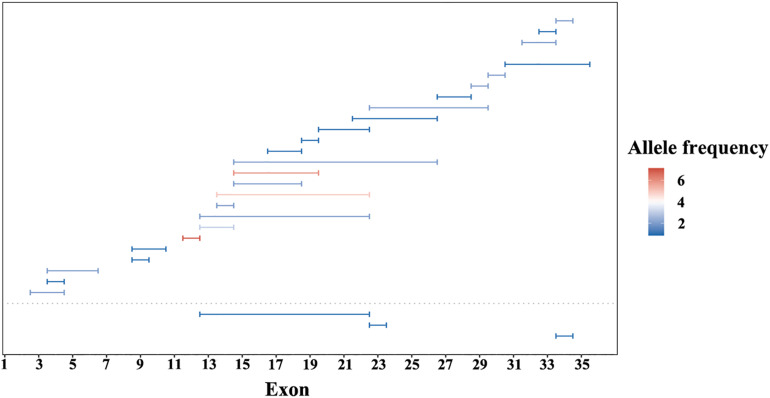
Copy number variations (CNV) of *EYS* gene in this study. CNV in G1 cohort under the dotted line and G2 cohort above. Lengths of the horizontal line represents the CNV range. Color from blue to red indicate an increase in allele frequency.

A summary of the genetic data from G1 and G2 revealed 262 variants among the 420 probands. The mutations included 76 frameshift, 65 missense, 59 nonsense, 31 CNV, 27 splicing change, and 4 non-frameshift indel mutations (Figure 1B). Most of these variants were clustered in exon 26 and exon 43. The most common pathogenic variant was a frameshift c.4957dupA (p.S1653Kfs^∗^2) in exon 26, with an allele frequency of 12.7% (107/841), followed by a c.8805C > A (p.Y2935X) variant in exon 43, with an allele frequency was 5.9% (50/841). Mutations in exons 4, 16, and 32 were also common, whereas the remaining variants were distributed across the whole gene. CNV was more common in exons 12–22 (Figure 2).

### Evaluation of Genotype–Phenotype Correlations

**FIGURE 3:**
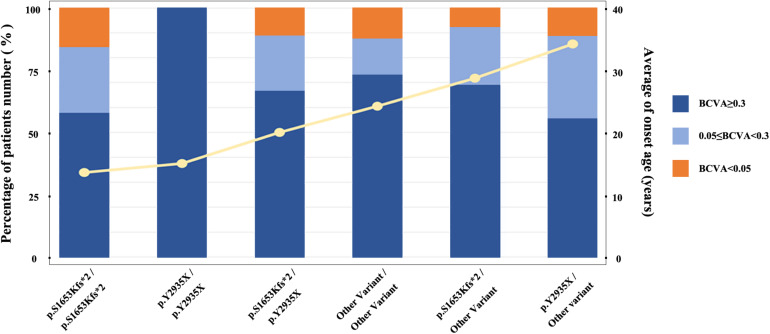
Relationship between average age at onset and specific causative mutation. The histogram displays the proportion distribution of three visual impairment levels, represented in different colors. The yellow line represents the average age at onset.

We stratified all patients into six groups to obtained a better assessment of the relationship between the genotypes of two mostly common pathogenic variants (M1: p.S1653Kfs^∗^2 and M2: p.Y2935X) and the *EYS*-IRD phenotypes (e.g., BCVA, and onset age). Grouped as follows: group I, 40 patients, carried only one of the two mutation hot spots, M1; group II, 24 patients, carried M1 homozygous mutation; group III, 16 patients, carried only one of the two mutation hot spots, M2; group IV, 7 patients, carried M2 homozygous mutation; group V, 18 patients, carried M1 and M2 compound heterozygosity mutation; group VI, 315 patients, without carried the above two common mutation. Comparison of the differences in age of onset and the severity of the loss of visual acuity of six groups and sorting the age of onset from left to right (Figure 3) revealed that group I and group IV had an earlier onset age than the other groups. No significant difference was noted in the proportion of patients with specific degrees of visual impairment in each group.

### Participants and Clinical Profiles in G1

In total, 39 affected *EYS*-IRD subjects were included in this study (G1 cohort); 30 were sporadic cases, 9 were ARRP, and all of them were Chinese. The clinical diagnosis was RP in 38 patients, and cone-rod dystrophy (CORD) in 1 patient (Supplementary Table 1). The average age of onset and age at first visit were 20.54 ± 11.33 (4–46) and 37.33 ± 11.36 (14–48) years, respectively. The average best corrected visual acuity (LogMAR) was 0.74 ± 1.06 (0–5) in this cohort. We identified the visual prognosis of *EYS*-IRD subjects by exploring the probability of low vision and blindness at different ages. The regression model was as follows: probability of low vision is Ln(P/1 − P) = 0.145 × age − 7.163 and probability of blindness is Ln (P/1 − P) = 0.098 × age − 6.56. When the cut-off probability was delimited as > 50%, the age of visual acuity when low vision and blindness were reached was 50 and 55 years.

**FIGURE 4:**
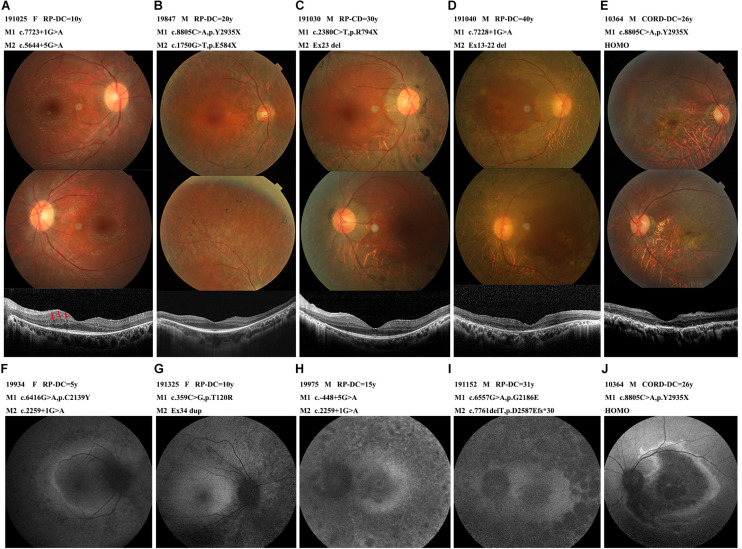
The photographs characteristics of the patients with different courses. **(A)** A 22-year-old female (191025) diagnosed with RP, showed RPE atrophic changes outside the vessel arcade without pigmentation, OCT with some cysts in the inner nuclear layer (arrow-heads). **(B)** A 44-year-old male (19847) diagnosed with RP, pigmentations were scattered in the peripheral retina. OCT showed relatively preserved macular structure. **(C)** A 43-year-old male (191030) diagnosed with RP, extensive choroid atrophy with minimal residual RPE outside the macular region. OCT showed ellipsoid and interdigitation zone discontinuity. **(D)** A 47-year-old male (191040) diagnosed with RP, showed the fundus appearance of the end-stage of the disease, widespread RPE and choroidal atrophy, bone spicules and vascular thinning were evident, macular affected. OCT showed thinning of the outer retina, ellipsoid completely disappeared, RPE atrophied with high reflection sediments. **(E,J)** A 31-year-old male (10364) diagnosed with CORD, marked atrophy of macular, OCT showed full retinal layers thinned, severe atrophy of the outer retina. Dark macular area and a hyperfluorescent ring within the vascular arcade in AF. **(F,G,H,I)** Autofluorescence images from early to late-stage disease. Hyper auto-fluorescent ring at the posterior pole in the early stages, evolve into low fluorescence in the whole field of view. Abbreviations: DC, disease course. HOMO, homozygosis. M, mutation.

All 38 probands with *EYS*-RP presented typical RP symptoms, with the most common initial symptom being nyctalopia. The visual field subsequently constricted and vision decreased. Lens opacities were present in 13 patients, with the youngest being 35 years old. No patients presented with nystagmus. Observation of the patients with different courses of disease by fundus photography revealed atrophy of the RPE in the peripheral retina and typical progression of RPE atrophy from the periphery to the macula, with disease aggravation and classic bone spicule pigmentation consistently appearing in the mid-peripheral retina; the macula was also sometimes affected in the later stages (Figure 4). The OCT images showed thinning of the outer retina, RPE atrophy with high reflection sediments, gradually reduction of the thickness of the ellipsoid and interdigitation zone until it completely disappeared from the peripheral region to the macular fovea, and a relative preservation of the retinal layers in the central macula. In patient 191,025, we noticed some cysts in the left OCT, located in the inner nuclear layer. Four *EYS*-RP probands underwent AF examination. The course of their disease ranged from 5 to 31 years, and they showed progression of abnormal fundus autofluorescence consistent with the fundus appearance (Figure 4). ERGs were all extinguished except in patient 19,562, whose ERGs showed a severe decrease in rod and cone function.

Patient 10,364, carrying homozygous c.8805C > A (p.Y2935X), was the only EYS-CORD patient. This patient was 31 years old and had experienced vision impairment at 5 years old, without nyctalopia. Fundus photography showed a marked atrophy of the macula and pigment deposition in the peripheral retina. His OCT shown thinning of the full retinal layers, disappearance of the ellipsoid zone, atrophy of the RPE layer with high granular reflection, and enhancement of the reflection bands of the choroid tissue. AF showed a dark macular area and a hyperfluorescent ring within the vascular arcade. The ERG showed a severely decreased amplitude (Figure 4).

## Discussion

The eyes shut homolog (*EYS*; OMIM: 612424) is a major disease-causing gene for autosomal recessive retinitis pigmentosa, and is mainly found in Chinese and Japanese populations (Pontikos et al., 2020), where it has a prevalence ratio of about 11% (Abd El-Aziz et al., 2010; Xiao et al., 2019) and 32.8% (Hosono et al., 2012; Iwanami et al., 2012; Arai et al., 2015; Yang et al., 2020), respectively. In this study, we performed a comprehensive analysis of the phenotype characteristics in the largest cohort to date of 39 Chinese *EYS* gene mutant patients. We also combined data from 381 patients from all over the world to summarize the mutation spectrum and genotype–phenotype correlations of the *EYS* gene.

The *EYS* gene was first reported to cause retinitis pigmentosa and associated phenotypes in 2008 (Abd El-Aziz et al., 2008; Collin et al., 2008). At present, at least 390 disease-causing mutations of the *EYS* gene have been reported, according to the HGMD database. In the present study, 42% (19/45) of the mutations in G1 cohort were novel, suggesting that the *EYS* gene mutation spectrum remains to be explored. This extensive mutation spectrum may provide a basis for more effective targeting for future gene therapy.

Mutations of c.6557G > A (p.G2186E) were the most common in the G1 cohort, with a frequency of 9%. This mutation has been reported as a hotspot in Chinese patients with RP (Gu et al., 2016). Previous studies have also identified c.6557G > A (p.G2186E) mutations as a major cause of ARRP in Japanese and Korean populations (Suto et al., 2014; Yoon et al., 2015; Yang et al., 2020), this mutation has also been detected in Dutch patients (Littink et al., 2010; Pierrache et al., 2019). In the present study, we discovered that 6.4% of the Chinese patients in this group displayed a c.1750G > T (p.E584X) mutation, and this seems to be a frequent mutation in the Chinese population. By contrast, the prevalence of this variant was lower in the other Asian ethnic groups, including Japanese and Korean patients (Yoon et al., 2015; Hirashima et al., 2017). Another reported causal mutation, c.7492G > C (p.A2498P), shared the same allele frequency of 6.4%, but has been identified only in Chinese populations. These two mutations, c.1750G > T (p.E584X) and c.7492G > C (p.A2498P), were only rarely mentioned in previous reports, probably due to the small sample sizes of the studies. In the present study, the larger sample size identified these two mutations as additional hotspots in Chinese populations. Two Japanese hotspot mutations, c.4957_4958insA and c.8805C > A (Numa et al., 2020), were detected less frequently in the G1 cohort, indicating differences between the hotspots among patients in the Asian population.

We detected *EYS* gene mutations of various types, including missense, nonsense, splicing, frameshift, CNV, and non-frameshift mutations, but the proportions of different mutation types were similar (Figure 1). The ratio of CNV identified in this study was 12%. The regions from exon 12 to exon 22 and the C-terminal part of the amino acid chain are prone to produce large deletion or duplication variants; however, CNV analysis and validation is a challenging task, as traditional sequencing technologies, such as PCR-based or target enrichment sequencing, have limitations in detecting CNV (Huang et al., 2017; Jin et al., 2018). Therefore, the proportion of CNV in the EYS gene may be underestimated. Compared with the other pathogenic genes of autosomal recessive IRD, CNV is a relatively common event in *EYS* gene (Pieras et al., 2011).

In this study, we have identified novel genotype–phenotype correlations among globally distributed patients with EYS-IRD. Although the analysis is limited by the case numbers and the amount of clinical information, these results demonstrated that the onset age of patients with *EYS* gene mutation might be affected by the specific mutation, but the overall average onset age of patients with *EYS* mutations is 21 years old. Compared with *USH2A*, the most common pathogenic gene of IRD, the onset of *EYS* patients occurs at a later age, and the visual acuity is preserved until the fifth decade in most patients (Huang et al., 2015).

Overall, the fundus of all the patients showed a relatively consistent phenotype. Our comparison of the fundus features of patients in different periods. In the early stage of the disease, the peripheral retinal pigment epithelium underwent extensive atrophy, the posterior pole was relatively preserved, the macular area of the fundus was generally normal, and the fovea reflection disappeared before 50 years of age. In the later stage of the disease, the macular area atrophied. Bone spicules showed increasing density with disease progression. The OCT of patient 191,025 showed some small cysts in the inner nuclear layer; these cysts were caused by Muller cell atrophy and necrosis in the inner core layer, as previously described (Makiyama et al., 2014; Mucciolo et al., 2018), but most patients do not show these cysts; they are not typical OCT characteristics of patients with *EYS* gene mutations.

The *EYS* gene mutations are mainly related to RP, but some subjects show autosomal recessive CORD (Pierrache et al., 2019; Tian et al., 2020). In our study, only one patient in the G1 cohort had a CORD diagnosis (an incidence of 2.6%). Our CORD patient carried a homozygous c.8805C > A (p.Y2935X) mutant, the same mutation identified in a Japanese CORD patient reported previously, who carried compound heterozygous variants c.8805C > A (p.Y2935X) and c.4957dupA (p.S1653Kfs^∗^2) (Katagiri et al., 2014). When compared with other *EYS*-RP patients, our CORD patient developed symptoms earlier, accompanied with vision damage. His fundus revealed a serve RPE dystrophy in the fovea; however, due to the relatively good condition of the posterior pole, the visual acuity of this patient was maintained at the same level as the other *EYS*-RP patients. Yang.et reported on 7 cases of CORD who carried c.8805C > A (p.Y2935X) heterozygous variant, the median onset age of these subjects was 40.0 (range 29.0–43.0), with a later age of onset compare with our CORD patient and other EYS-RP cohort. The fundus of all EYS-CORD patients showed earlier progressive and more severe RPE dystrophy in the fovea than peripheral retina, unanimously (Yang et al., 2020). Unfortunately, the reasons for the identical genotypes associated different phenotypes in both RP and CRD remain unclear.

## Conclusion

In conclusion, our analysis of the extensive global data from 420 IRD patients and 262 distinct variants demonstrated the characteristic genotypes and phenotypes and mutation spectra with hotspots in the *EYS* gene. Our findings provide critical information for clinical genetic diagnosis, as well as for therapeutic management of EYS-associated retinal disease.

## Data Availability Statement

The original contributions presented in the study are included in the article/Supplementary Material, further inquiries can be directed to the corresponding author/s.

## Ethics Statement

The studies involving human participants were reviewed and approved by Institutional Medical Ethics Committee. Written informed consent to participate in this study was provided by the participants’ legal guardian/next of kin.

## Author Contributions

Z-BJ designed and supervised whole study. KX and D-FC collected the data and interpreted the results. HC, R-JS, HG, X-FW, Z-KF, XZ, and YX conducted the data analysis. KX wrote the manuscript. YL and Z-BJ revised the manuscript. All authors contributed to the article and approved the submitted version.

## Conflict of Interest

The authors declare that the research was conducted in the absence of any commercial or financial relationships that could be construed as a potential conflict of interest.

## Footnotes

1 https://sph.uth.edu/retnet/

2 http://www.retinogenetics.org/

## Supplementary Material

Supplementary Figure 1 Family pedigrees and identified EYS variations for G1 cohort. The generation number and *EYS* genotypes for probands and their family members are demonstrated under their symbols. The Black filled square s (male) and circles (female) represent the affected patients, and unaffected family members are represented by unfilled icons. The slash symbol indicates deceased member. Probands are marked by arrows.
