# Supplementary figures and images for: Genotype Profile of Global EYS-Associated Inherited Retinal Dystrophy and Clinical Findings in a Large Chinese Cohort
Source: Front Cell Dev Biol. 2021 Jun 11;9:634220. doi: 10.3389/fcell.2021.634220 (PMC8226124; doi:10.3389/fcell.2021.634220)

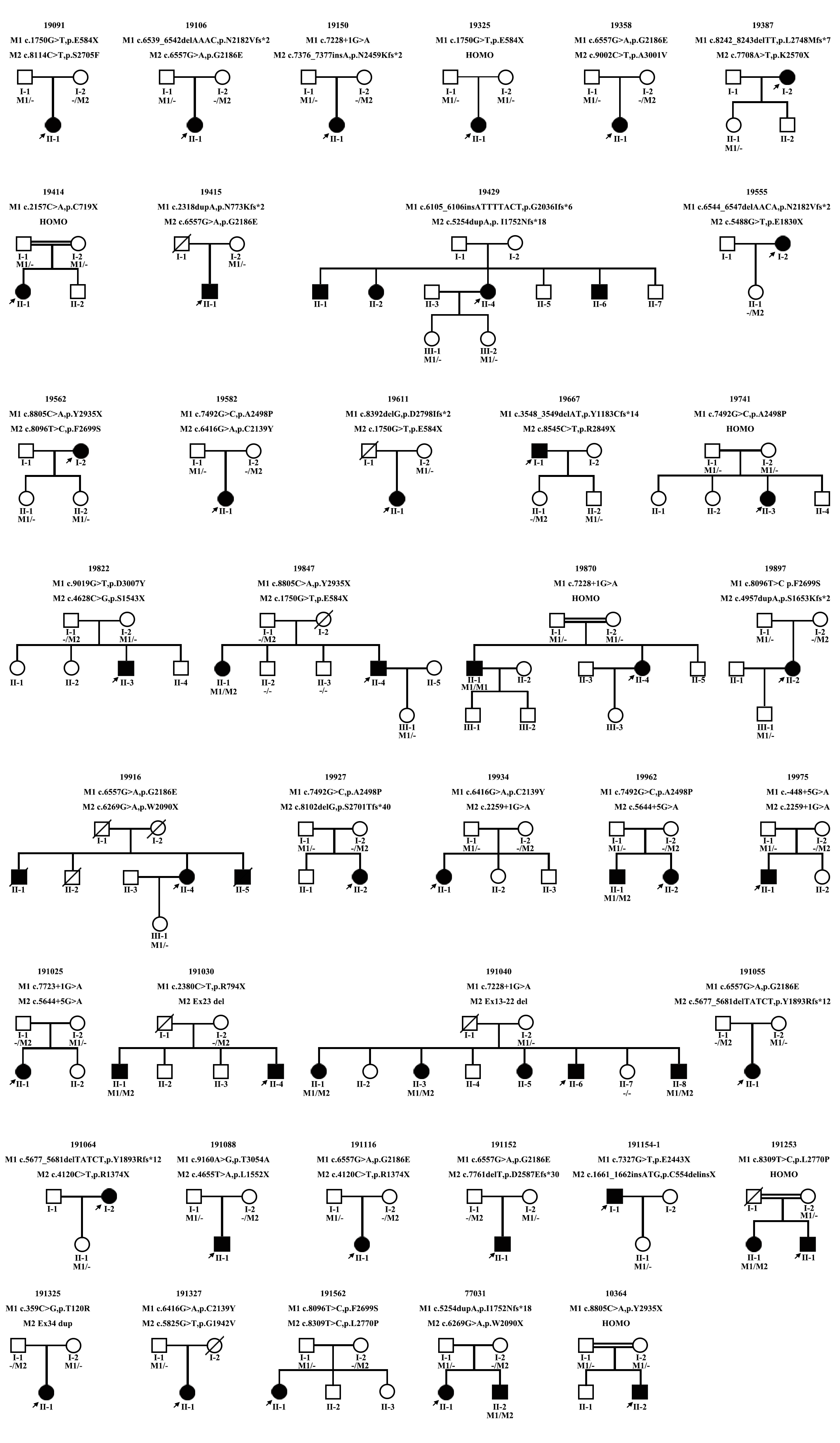

Supplement: Supplementary Figure 1 — Family pedigrees and identified EYS variations for G1 cohort. The generation number and EYS genotypes for probands and their family members are demonstrated under their symbols. The Black filled square s (male) and circles (female) represent the affected patients, and unaffected family members are represented by unfilled icons. The slash symbol indicates deceased member. Probands are marked by arrows. [file Image_1.TIF]
